# Cerebellar Network Compensation in Parkinson’s Disease: Functional Connectivity Across Motor and Cognitive Circuits

**DOI:** 10.64898/2026.05.19.26352927

**Published:** 2026-05-22

**Authors:** Chi-Ying R. Lin, Thamires N.C. Magalhães, Shayla S. Yonce, Ihika Rampalli, Rory Mahabir, Jessica A. Bernard, the Parkinson’s Progression Markers Initiative (PPMI)

**Affiliations:** 1 Alzheimer’s Disease and Memory Disorders Center, Department of Neurology, Baylor College of Medicine, Houston TX; 2 Parkinson’s Disease Center and Movement Disorders Clinic, Department of Neurology, Baylor College of Medicine, Houston TX; 3 Department of Psychological and Brain Sciences, Texas A&M University, College Station, TX; 4 Texas A&M Institute for Neuroscience, Texas A&M University, College Station, TX

**Keywords:** Parkinson’s, cerebellum, functional connectivity, disease duration, age

## Abstract

**Introduction.:**

The cerebellum is increasingly recognized as a key contributor to cognitive reserve and network adaptation in Parkinson’s disease (PD). However, how cerebello–cortical and cerebello–basal ganglia connectivity reorganizes across disease duration and cognitive status remains incompletely understood.

**Methods.:**

Resting-state fMRI data from the Parkinson’s Progression Markers Initiative were analyzed in 172 individuals with PD. We investigated cerebello-basal ganglia and cerebello-cortical connectivity using ROI-to-ROI and seed-to-voxel pipelines respectively, providing novel insights into both subcortical and cortical effects. Effects of age, disease duration, cognitive status, motor symptom severity, and dopaminergic medication were assessed.

**Results.:**

Across all participants, cerebellar lobule VI and vermis VI showed robust positive connectivity with the pallidum, along with high intra-cerebellar coupling. When controlling for dopaminergic medication, lobule V connectivity with the primary motor cortex was reduced. Age was associated with lower cerebello–basal ganglia connectivity widespread across nodes, evident across medication states. Disease duration showed region-specific effects: in cognitively normal PD, longer duration corresponded to stronger lobule V–temporal cortex connectivity and higher Crus I–precentral gyrus connectivity than PD with cognitive dysfunction. Motor symptom severity was not related to connectivity.

**Conclusions.:**

Cerebellar connectivity patterns in PD are linked to disease duration and cognitive preservation. Enhanced cerebello–cortical coupling in cognitively normal PD may reflect compensatory network recruitment that diminishes with cognitive decline. These findings position cerebellar networks as candidate markers of compensatory capacity and disease trajectory in PD.

## Introduction

Parkinson’s disease (PD) is the second most common neurodegenerative disorder, affecting more than 10 million people worldwide.^1^ It is traditionally defined by its hallmark motor symptoms, including bradykinesia, rigidity, tremor, and postural instability, resulting from dopaminergic cell loss in the substantia nigra pars compacta. However, the disease extends far beyond motor dysfunction: cognitive impairment is a particularly distressing non-motor feature, with cumulative prevalence up to 50% of PD individuals eventually developing dementia within 10 years of the diagnosis and 80% after 20 year of diagnosis.^2^ Importantly, the trajectory from cognitively normal PD to mild cognitive impairment and dementia is highly variable,^3^ suggesting that compensatory mechanisms and brain reserve play a significant role in modulating the manifestations of non-motor features of PD.

Despite accounting for only close to 10% of intracranial volume, the cerebellum houses the majority of the brain’s neurons.^4-6^ Over the past several decades, the cerebellum been recognized as an important hub to maintain cognitive function, supporting executive function, language, and working memory, in addition to its classically recognized contributions to motor behavior.^7,8^ Additionally, the cerebellar regions that are most strongly implicated in cognition also maintain robust connections with motor and basal ganglia circuits, positioning the “cognitive cerebellum” as an interface between motor and higher-order networks in PD.^9^ Prior studies exhibited significant cerebellar network alterations in Parkinson’s disease and identifies their co-occurrence with cerebellar atrophy.^9-13^ Additionally, non-linear progression of cerebellar microstructural changes in PD was identified, reflecting adaptive reorganization concurrent with ongoing striatal dopaminergic decline.^14^ Importantly, mirroring observations in Alzheimer’s disease, the cerebellum is largely preserved from direct early PD pathology, retaining its structural integrity and showing enhanced or reorganized functional connectivity.^15,16^ Such preservation implies that the cerebellum may provide a compensatory substrate that supports *network-level adaptation* across the trajectory of PD.^9,17-19^ These findings overall support the hypothesis that the cerebellum can contribute to neural scaffolding and reserve in aging and neurodegenerative diseases.^7,20-24^ Despite this, the role of cerebellar network changes in PD disease trajectory remains underexplored.

In the present study, we investigate whether cerebellar functional connectivity provides compensatory support for cognition and motor-related networks across the spectrum of PD. Using resting-state fMRI from the Michael J. Fox Foundation Parkinson’s Progression Markers Initiative,^16^ we examine the functional connectivity between cerebellar lobules and both cerebral cortical and subcortical regions. In particular, we focused on subcortical connections between the cerebellum and basal ganglia, given the role of these circuits in PD pathophysiology. Additionally, prior work in aging has demonstrated functionally segregated topographic motor and cognitive cerebellar-basal ganglia circuits, which are relevant for behavior and impacted in advanced age, likely reflecting age-related dopamine decline.^25^ These same circuits are modulated by dopaminergic manipulation in both healthy adults and individuals with PD, underscoring their clinical relevance across disease progression.^10,26^ We hypothesized that cerebellar functional connectivity with cortical and basal ganglia networks is altered in PD, reflecting compensatory responses to dopaminergic decline acting on age-related circuit vulnerability.

## Methods

Data used in the preparation of this article was obtained on January 2, 2025 from the Parkinson’s Progression Markers Initiative (PPMI) database (www.ppmi-info.org/access-data-specimens/download-data), RRID:SCR_006431.^27^ For up-to-date information on the study, visit www.ppmi-info.org. This analysis used data openly available from PPMI. Demographic data was selected from the “Participant Status, Demographics, Socio-Economic, and PD Diagnosis History” files. Entries were selected by filtering for patients designated as controls and having PD per the PD Diagnosis History file. For each patient, baseline data was selected. Only participants with MRI and fMRI were included in the study. Cerebellar coverage was evaluated by two trained lab members (TM and MM) and independently verified by the senior author (JAB). Participants were excluded if cerebellar imaging did not extend below Crus I. A total of 172 PD participants were then included. Demographic and clinical covariate data included age, sex, education (years), disease duration (years), Montreal Cognitive Assessment (MoCA) score, International Parkinson’s and Movement Disorders Society Unified Parkinson's Disease Rating Scale (MDS-UPDRS) part III score, and PD medication history. The dopaminergic medication was stratified into three categories for statistical investigation: i) carbidopa/levodopa (regular or extended release), ii) non-carbidopa/levodopa dopaminergic medication (amantadine, selegiline, rasagiline, ropinirole, pramipexole), and iii) drug naïve. With a total score of 132, the MDS-UPDRS was stratified into mild (32 ≤ below), moderate (33-58), and severe (≥ 59) to represent different level of motor impairment severity. Furthermore, from the cognitive status point of view, MoCA > 26 was categorized as PD with normal cognitive function, and MoCA < 26 is PD with cognitive dysfunction.^28^

### Neuroimaging Data Processing and Analysis

All data processing followed procedures used in our recent work.^29^ For the sake of clarity, consistency, and replication, we have restated those here using boilerplate text with additional details about choices made that were specific to this data set. All data were pre-processed using the CONN toolbox (22.v2407).^30^ Functional and anatomical data were preprocessed using a flexible preprocessing pipeline including realignment, outlier detection, direct segmentation and MNI-space normalization, and smoothing.^31^ Functional data were realigned using the SPM realign & unwarp procedure,^32^ where all scans were co-registered to a reference image (first scan of the first session) using a least squares approach and a 6 parameter (rigid body) transformation,^33^ and resampled using b-spline interpolation. However, we did not incorporate slice timing correction given the nature of the data analyzed here. Because this data set includes scans collected across multiple sites, the inclusion of re-slicing during realignment could result in inconsistencies and errors in processing, and this allows for more reliable motion parameter estimation. This also avoids challenges and inaccuracies that would result from incorrect slice-order assumptions. Potential outlier scans were identified using ART as acquisitions with subject motion above 0.9 mm or global BOLD signal changes above 5 standard deviations,^30,31,34^ which is the equivalent of the “intermediate” settings as defined by CONN. A reference BOLD image was computed for each subject by averaging all scans excluding outliers. Functional and anatomical data were normalized into standard MNI space, segmented into grey matter, white matter, and CSF tissue classes, and resampled to 2 mm isotropic voxels following a direct normalization procedure using SPM unified segmentation and normalization algorithm with the default tissue probability map template.^31,35-37^ Last, functional data were smoothed using spatial convolution with a Gaussian kernel of 8 mm full width half maximum (FWHM).

Functional data were denoised using a standard denoising pipeline,^31^ including the regression of potential confounding effects characterized by white matter timeseries (5 CompCor noise components), CSF timeseries (5 CompCor noise components), motion parameters and their first order derivatives (12 factors),^38^ outlier scans (12 factors),^34^ and linear trends (2 factors) within each functional run, followed by bandpass frequency filtering of the BOLD timeseries between 0.008 Hz and 0.09 Hz.^39^ This was applied to the resting state data in order to focus on slow-frequency fluctuations while minimizing the influence of physiological, head-motion and other noise sources.^39^ Potential confounding effects to the estimated BOLD signal were determined and removed separately for each voxel and for each subject and functional run/session, using Ordinary Least Squares (OLS) regression to project each BOLD signal timeseries to the sub-space orthogonal to all potential confounding effects using an anatomical component-based noise correction procedure (aCompCor).^40^

All statistical analyses of resting-state functional connectivity were also completed in CONN. Data was analyzed using a targeted ROI-to-ROI approach in order to focus on the cerebello-basal ganglia circuit. This was then followed by exploratory whole brain analyses of our cerebellar regions of interest to better understand differences in cerebellar-whole brain connectivity associated with cognitive decline in PD. For ROI-to-ROI analyses, cerebellar regions included bilateral hemispheric lobules I–IV, V, VI, Crus I, Crus II, and corresponding vermal regions (vermis VI, vermis Crus I, and vermis Crus II). All regions were as defined by the SUIT atlas. Subcortical ROIs included bilateral caudate, putamen, pallidum, and accumbens using the atlas-based ROIs available in CONN.^41,42^ We first established patterns of cerebello-basal ganglia connectivity in all PD patients, and further investigated impacts of age, disease duration, and motor symptom severity as indexed by the MDS-UPDRS. Connectivity results were considered significant at a connection-level threshold of *p < 0.05* and a cluster-level threshold of *pFDR < 0.05* (MVPA omnibus test). For seed-to-voxel analyses, we focused on Lobule V and Crus I in the right hemisphere to reduce multiple comparison burden and to serve as a targeted initial examination of cerebellar connectivity patterns as we explored differences in those with and without cognitive decline. Prior work looking at both the left and right cerebellar hemispheres has demonstrated comparable patterns of connectivity in these seeds;^43-45^ as such, we chose to focus on the right hemisphere. All whole-brain analyses were evaluated using threshold-free cluster enhancement (TFCE),^46^ a robust statistical approach implemented using 1,000 permutations. Like in the ROI-ROI analyses, we also investigated effects of disease duration and motor symptom severity. These seed-based analyses were implemented as supplementary validation steps to confirm the expected network topology and connectivity patterns.

## Results

The 172 participants with PD had an average age of 65.0 ± 7.8 years, including 60 women and 112 men. They had 16.6 ± 3.7 years of education, a MoCA score of 26.9 ± 2.4, and a MDS-UPDRS Part III score of 22.1 ± 10.3. Disease duration was 49.3 ± 37.6 months. Based on MoCA scores, 105 participants were cognitively normal (MoCA ≥ 26), whereas 67 showed cognitive dysfunction (MoCA < 26; 58 with MoCA 23-26, and 9 with MoCA ≤ 22). With respect to medication status, 68 participants were treated with carbidopa/levodopa, 64 received non-carbidopa/levodopa dopaminergic medications, and 40 were medication-naïve. Disease severity, assessed using the Hoehn and Yahr (H&Y) scale, was distributed as follows: 62 participants were in stage 1, 58 in stage 2, and 52 in stage 3.

### Foundational cerebellar network alterations in PD

**Figure 1.**
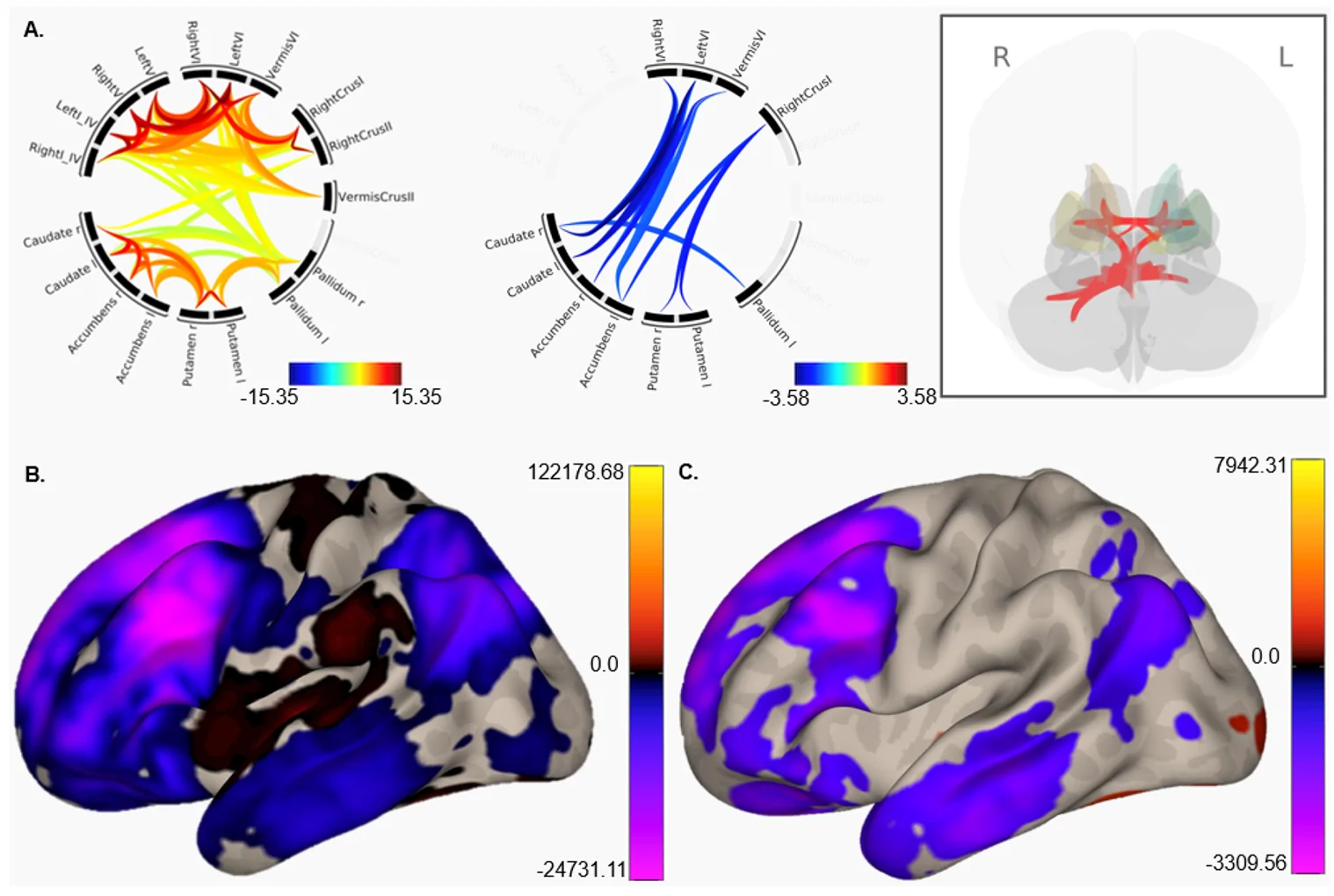
Foundational cerebellar network alterations in Parkinson’s disease. A. Connectome rings showing the cerebello-basal ganglia circuits in all PD patients, while controlling for dopaminergic medication. The hot colors (left) represent positive correlations between ROIs while the cool colors (right) show negative correlations. The inset (gray box) includes a 3D visualization of the positive associations between ROIs within and between the cerebellum and basal ganglia. Color bars indicate the strength of the relationships between ROIs. R: right; L: left. B. Connectivity between cerebellar Lobule V and the cortex in all PD patients demonstrating positive associations with motor and premotor cortical regions, and anti-correlations with frontal, temporal, and parietal cortices, consistent with past findings in healthy young and older adults. C. When controlling for dopaminergic medication, the pattern of Lobule V-cortical connectivity is lessened, and the associations with motor cortical regions are no longer significant. Chord diagrams depict the magnitude and direction of age-related associations. All results are thresholded at *p < 0.05* and cluster-level *pFDR < 0.05* (MVPA omnibus test). Color bars for B and C represent the significant connectivity patterns, as indexed by TFCE values.

We first asked whether foundational cerebellar network alterations could be detected in PD regardless of cognitive function and while controlling for dopaminergic medication (Figure 1). Using our ROI-ROI approach to investigate cerebellar-basal ganglia connectivity, we found patterns of both positive and negative connectivity (anticorrelations) between cerebellar and basal ganglia regions. The complete results are detailed in Table 1. Of note, across all PD participants, robust positive connectivity was identified between the globus pallidus and both right cerebellar lobule VI (lobule VI – pallidum) and vermis VI (vermis VI – pallidum) (Figure 1A). Lobules I-IV, V, and VI also showed positive associations with the putamen, reflecting the established motor (i.e., lobule I-V) and cognitive (i.e., lobule V and VI) dissociation within the cerebello-striatal circuit, as well as robust intra-cerebellar connections (Table 1, Figure 1A). These results suggest that cerebellar circuits may be upregulated to compensate for basal ganglia dysfunction in PD. When investigating whole brain connectivity, Lobule V demonstrated the expected canonical pattern of positive correlations with motor cortical regions and anti-correlations with frontal and association areas, supporting the validity of the connectivity pipeline (Table 2, Figure 1B).^47-49^ A comparable connectivity pattern was observed for Crus I, reflecting its well-established canonical role within the frontal association network. However, when controlling for dopaminergic medication, Lobule V connectivity with primary motor cortex (M1) was no longer present across all PD participants (Table 2, Figure 1C), and there were no group differences identified when comparing cognitively normal PD participants to those with cognitive dysfunction. This suggests that medication may modulate cerebello-cerebral motor coupling, regardless of cognitive capacity. There were no subgroup differences observed for Crus I, and medication effects were less pronounced relative to Lobule V. The overall connectivity pattern of canonical Crus I network remained intact, though less extensive.

### Effect of age

**Figure 2.**
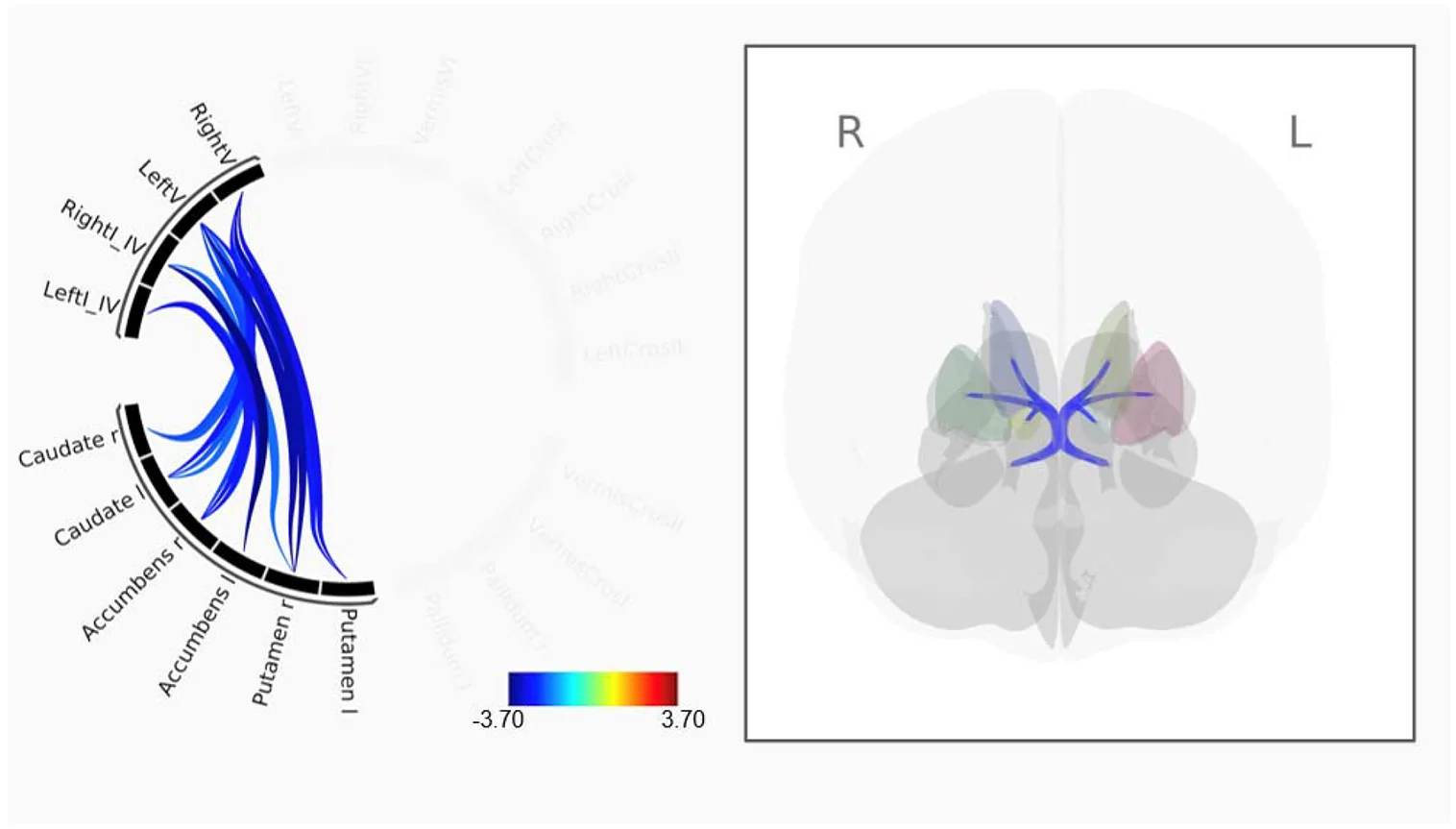
Age-related alterations in cerebellar–basal ganglia connectivity. Age is negatively associated with cerebello—basal ganglia connectivity across individuals with PD. Left: connectome ring showing lower connectivity (anticorrelations) with greater age between regions of the cerebellum and basal ganglia, consistent with past work. The inset (gray box) includes a 3D visualization of these associations between the cerebellum and basal ganglia. The color bar indicates the strength of the relationships between ROIs. Chord diagrams depict the magnitude and direction of age-related associations. All results are thresholded at *p < 0.05* and cluster-level *pFDR < 0.05* (MVPA omnibus test). PD: Parkinson’s disease.

We further studied whether the effect of age impacts cerebellar functional connectivity in PD, controlling for the effect of dopaminergic medication. We found that in the full PD cohort, age was negatively associated with widespread functional connectivity between cerebellar lobules and all regions of basal ganglia (Table 3, Figure 2), consistent with patterns seen in healthy older adults as well.^25^ There are consistent age-related anti-correlations involving the pallidum, putamen, and caudate, as well as cerebellar lobules V, VI, and Crus I/II. However, there were no statistically significant group differences between cognitively normal PD and PD with cognitive dysfunction in the relationships between cerebello-basal ganglia connectivity and age.

### Effect of disease duration

**Figure 3.**
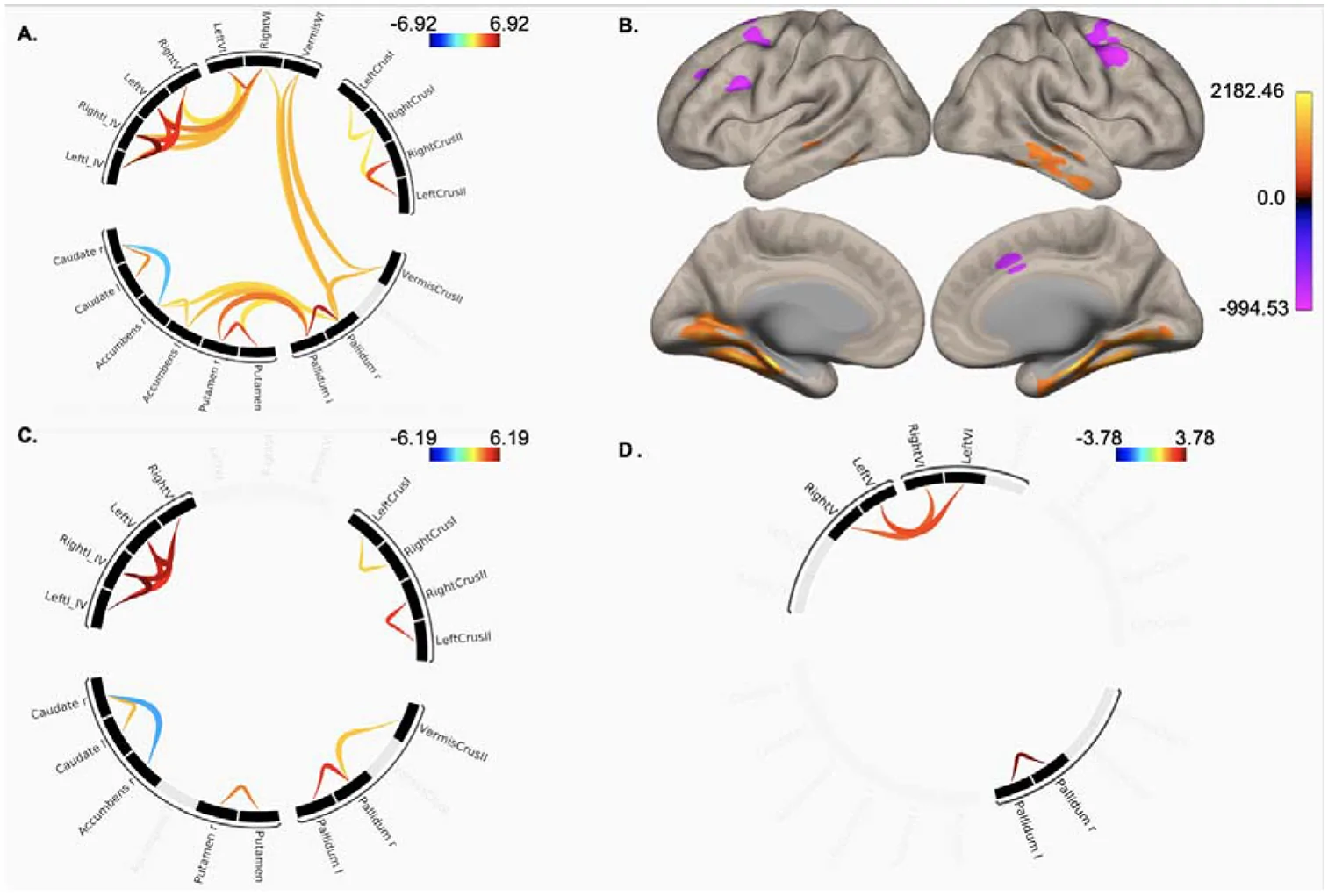
Disease duration effects on cerebellar–cortical connectivity. A. Associations between connectivity and disease duration in the entire PD sample largely demonstrating patterns of within structure (cerebellum and basal ganglia) connectivity as disease duration lengthens. B. Exploratory whole-brain analyses of Lobule V connectivity associations with disease duration. Connectivity with medial temporal lobe regions was increased with longer disease duration, while it was decreased with premotor and prefrontal areas. C. Connectome ring demonstrating associations between cerebello-basal ganglia connectivity and disease duration in cognitively normal PD only, and D. PD decline. There are no significant group differences between the cognitively normal and cognitive decline groups; however, qualitatively, the associations with disease course are fewer in the cognitive decline group. Color bars are indicative of connectivity strength. For panel B, numbers indicate TFCE values. Maps are thresholded at a connection-level *p < 0.05* and cluster-level *pFDR < 0.05* (MVPA omnibus test). PD: Parkinson’s disease.

**Figure 4.**
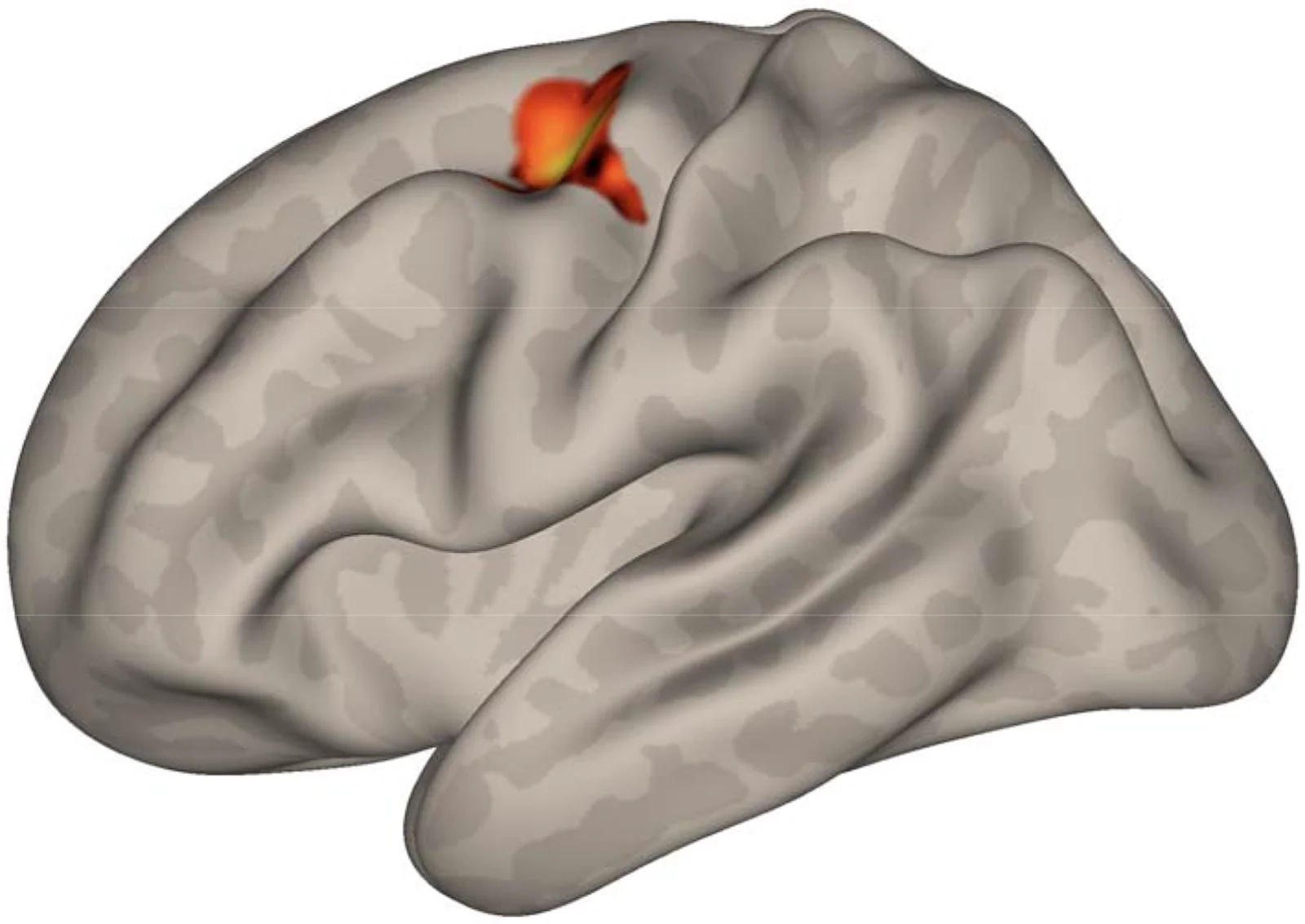
Cognitive-group differences in Crus I–precentral gyrus connectivity. Cognitively normal PD participants demonstrated higher Crus I–left precentral gyrus connectivity than PD-MCI and PDD participants, even after controlling for disease duration. This connectivity pattern aligns with preserved cerebellar–cortical support for cognition in early PD. Color bar indicates strength of connectivity as indexed by TFCE values. Results are thresholded at *p < 0.05* and cluster-level *pFDR < 0.05* (MVPA omnibus test). PD: Parkinson’s disease; PD-MCI: PD with mild cognitive impairment; PDD: Parkinson’s disease dementia.

We next investigated whether disease duration can impact cerebellar functional connectivity in PD. When looking first at the ROI-ROI, longer disease duration was associated with robust positive connectivity between right and vermis lobule VI and the right pallidum, in addition to within cerebellar and basal ganglia connectivity (Figure 3A; Table 4). However, while there were no significant group differences when comparing cognitively normal PD participants relative to those with cognitive dysfunction, there are notable qualitative differences. Within the cognitively normal sample, there are several connections within both the basal ganglia and cerebellum, but these are far fewer in those with cognitive decline (Figure 3C-D, Table 4). When exploring these associations in whole-brain seed-to-voxel analyses, in cognitively normal PD, longer disease duration was robustly associated with stronger Lobule V – middle and inferior temporal gyrus connectivity, while there were negative associations with premotor and prefrontal regions (Figure 3B, Table 5). Furthermore, the group-level analyses showed that cognitively normal PD participants exhibited higher Crus I – left precentral gyrus connectivity compared to cognitive decline participants, even after controlling for disease duration (Figure 4) (coordinates: −28, −14, 52, cluster size=571 voxels, TFCE=966.85, peak p-FEW=.018). This pattern suggests that cerebello–cortical connectivity from Crus I to motor cortical regions, which is not typically a part of the Crus I network,^49^ may be a source of compensation in PD for those without cognitive decline. There were no associations with Crus I when looking at the full PD sample.

### Association with motor symptom severity

We additionally examined whether motor symptom severity, measured by the MDS-UPDRS Part III, was associated with cerebellar functional connectivity. Across all analyses, no significant associations emerged between motor severity and any connectivity measures, regardless of whether dopaminergic medication status was controlled. These results indicate that clinical motor symptom burden did not systematically relate to cerebellar network alterations within this cohort.

## Discussion

Our findings provide strong support for the compensatory role of the cerebellum in PD even in the presence of cognitive decline. Recent conceptualizations of the cerebellum in aging have suggested that it provides critical scaffolding for cortical function in aging, in the face of age-related structural and functional declines.^20,50,51^ This parallels the concept of cerebellar reserve wherein relatively preserved cerebellar resources may compensate in the face of neurodegenerative disease.^24,52^ Our present study has provided further support for this framework in PD. Enhanced cerebello-precentral gyrus and cerebello-pallidal connectivity in cognitively normal PD suggests that the cerebellum is recruited to scaffold cognition during early disease stages.^19^ The concurrent involvement of motor (precentral gyrus, pallidum) and association cortices underscores that cerebellar compensation in PD is not confined to “cognitive” circuits but instead spans integrated motor–cognitive networks that support goal-directed behavior. These results are consistent with theories of cognitive reserve and neural scaffolding,^24,52-54^ which posit that the brain adapts to pathology by engaging alternative networks. Importantly, we showed that purportedly compensatory cerebellar connectivity relates to disease duration and cognitive status, even in the context of widespread age-related declines in cerebello–basal ganglia coupling. In addition, the absence of significant associations between MDS-UPDRS motor severity and cerebellar connectivity suggests that these compensatory processes are not directly linked to clinical motor burden, but rather reflect network-level adaptations that may operate independently of motor symptom severity.

With longer disease duration, however, compensatory mechanisms decline, as evidenced by reduced cerebello-cortical connectivity in association networks. This may suggest that cerebellar reserve has limits, and once exceeded, patients progress more rapidly toward dementia; however, longitudinal investigations are critical for investigating this more directly. These findings underscore the dynamic nature of cerebellar contributions, with compensatory network engagement evident in cognitively normal PD but diminished as cognitive dysfunction emerges. Importantly, our disease-duration analyses also revealed that in cognitively normal PD, Lobule V connectivity with middle and inferior temporal cortex was higher with longer disease duration, consistent with targeted, region-specific recruitment that may help sustain cognitive function despite advancing pathology. These regions are outside the canonical Lobule V motor network.^47-49^ In contrast, only a small cerebellar cluster showed disease-duration effects in the cognitive-decline group, further supporting the notion that compensatory capacity diminishes as cognitive impairment emerges.

Our findings also resonate with broader work on aging-related cerebellar and dopaminergic decline. The spatial distribution and magnitude of the anti-correlations in our study findings closely mirrored previously reported cerebello–basal ganglia patterns observed in older adults, with aging in PD characterized by reduced synchrony between cerebellar and striatal nodes.^25^ Seidler and colleagues reviewed evidence showing that older adults experience structural atrophy of the cerebellum alongside degeneration of dopaminergic systems, especially within the nigrostriatal pathway.^20^ These changes contribute to motor slowing, postural instability, and reduced coordination, even in otherwise healthy aging. Importantly, such dopaminergic denervation places the aging brain on a continuum with preclinical Parkinson’s disease.^20^ The parallel vulnerability of both cerebellar and basal ganglia circuits suggests that the altered cerebellar connectivity we observed in Parkinson’s disease may, in part, reflect an amplification of mechanisms already present in aging. This convergence highlights the cerebellum’s integral role in compensatory processes and circuits but also underscores its susceptibility when dopaminergic inputs are diminished. Thus, our study extends prior work by demonstrating how basal ganglia–cerebellar interactions may shape the trajectory from normative aging to Parkinsonian pathology, with and without cognitive decline. Of note, our medication-controlled analyses revealed reduced Lobule V – M1 connectivity across PD subgroups, suggesting that dopaminergic medication is associated with cerebello–motor coupling; however, there were no group differences, indicating broadly similar dopaminergic medication sensitivity across cognitive subtypes.

Clinically, the observation that cerebellar connectivity shows disease-duration–dependent and Crus I–motor cortical coupling in cognitively normal Parkinson’s disease, but not in those with cognitive decline, suggests that cerebellar networks may reflect compensatory capacity that is not captured by standard clinical scales, supporting their potential relevance as imaging biomarkers and neuromodulatory targets (e.g., transcranial magnetic stimulation) to serve as a promising avenue to sustain compensatory capacity. However, several limitations temper these implications. The cross-sectional, resting-state fMRI design yields correlational measures and precludes inference about longitudinal trajectories of cerebellar compensation or decline, underscoring the need for replication in prospective cohorts integrating task-based and structural imaging. Clinical classification schemes, including MoCA cutoffs and MDS-UPDRS severity categories, may not fully capture the complexity of cognitive and motor progression in PD, potentially obscuring more subtle brain–behavior relationships and contributing to the limited connectivity differences observed across UPDRS-based severity. Finally, because participants were grouped primarily by cognitive status rather than detailed motor phenotypes, future work should incorporate more granular composite clinical indices that integrate both motor and non-motor progression to better disentangle domain-specific versus shared cerebellar compensatory mechanisms over time.

## Conclusion

In summary, cerebellar functional connectivity shows dynamic, region-specific alterations across the cognitive stages of Parkinson’s disease. Several enhanced connectivity patterns in those that are cognitively normal, and in an earlier more intact cognitive stage are suggestive of compensatory support. These results highlight cerebello-basal ganglia and –cortical connectivity as a promising neuroimaging biomarker of cognitive disease progression and treatment response, and underscore the need to determine how preserved or strengthened cerebellar pathways can be harnessed for therapeutic development, including targeted neuromodulation.

## Figures and Tables

**Table 1.** Region of interest (ROI) to ROI analyses for the cerebello-basal ganglia functional network across the full Parkinson’s disease cohort.

	Region 1	Region 2	Statistic	p-unc	p-FDR
**Positive**	Right VI	Left VI	T(170) = 15.35	< 0.0001	< 0.0001
	Vermis VI	Right VI	T(170) = 12.52	< 0.0001	< 0.0001
	Vermis VI	Left VI	T(170) = 11.99	< 0.0001	< 0.0001
	Right I to IV	Left I to IV	T(170) = 13.77	< 0.0001	< 0.0001
	Right V	Left V	T(170) = 13.61	< 0.0001	< 0.0001
	Left V	Left I to IV	T(170) = 12.14	< 0.0001	< 0.0001
	Right V	Right I to IV	T(170) = 12.05	< 0.0001	< 0.0001
	Right V	Left I to IV	T(170) = 10.70	< 0.0001	< 0.0001
	Left V	Right I to IV	T(170) = 10.37	< 0.0001	< 0.0001
	Right Crus I	Left Crus I	T(170) = 14.73	< 0.0001	< 0.0001
	Left Crus II	Left Crus I	T(170) = 14.31	< 0.0001	< 0.0001
	Right Crus II	Right Crus I	T(170) = 13.84	< 0.0001	< 0.0001
	Left Crus II	Right Crus II	T(170) = 12.67	< 0.0001	< 0.0001
	Left Crus II	Right Crus I	T(170) = 9.70	< 0.0001	< 0.0001
	Right Crus II	Left Crus I	T(170) = 9.56	< 0.0001	< 0.0001
	Right putamen	Left putamen	T(170) = 12.08	< 0.0001	< 0.0001
	Right caudate	Left caudate	T(170) = 11.79	< 0.0001	< 0.0001
	Right caudate	Right accumbens	T(170) = 10.75	< 0.0001	< 0.0001
	Left caudate	Right putamen	T(170) = 9.06	< 0.0001	< 0.0001
	Right accumbens	Left accumbens	T(170) = 8.99	< 0.0001	< 0.0001
	Left caudate	Left accumbens	T(170) = 8.66	< 0.0001	< 0.0001
	Left caudate	Right accumbens	T(170) = 7.93	< 0.0001	< 0.0001
	Right accumbens	Right putamen	T(170) = 7.31	< 0.0001	< 0.0001
	Left accumbens	Right putamen	T(170) = 6.61	< 0.0001	< 0.0001
	Left accumbens	Left putamen	T(170) = 6.12	< 0.0001	< 0.0001
	Right caudate	Left accumbens	T(170) = 6.20	< 0.0001	< 0.0001
	Left caudate	Left putamen	T(170) = 6.05	< 0.0001	< 0.0001
	Right caudate	Right putamen	T(170) = 5.51	< 0.0001	< 0.0001
	Right caudate	Left putamen	T(170) = 5.45	< 0.0001	< 0.0001
	Right accumbens	Left putamen	T(170) = 5.04	< 0.0001	< 0.0001
	Left VI	Left V	T(170) = 13.44	< 0.0001	< 0.0001
	Right VI	Right V	T(170) = 13.24	< 0.0001	< 0.0001
	Left VI	Right V	T(170) = 11.23	< 0.0001	< 0.0001
	Vermis VI	Right V	T(170) = 10.63	< 0.0001	< 0.0001
	Right VI	Left V	T(170) = 9.49	< 0.0001	< 0.0001
	Vermis VI	Left V	T(170) = 8.66	< 0.0001	< 0.0001
	Right VI	Right I to IV	T(170) = 8.29	< 0.0001	< 0.0001
	Left VI	Left I to IV	T(170) = 7.64	< 0.0001	< 0.0001
	Vermis VI	Right I to IV	T(170) = 7.22	< 0.0001	< 0.0001
	Left VI	Right I to IV	T(170) = 7.00	< 0.0001	< 0.0001
	Right VI	Left I to IV	T(170) = 6.83	< 0.0001	< 0.0001
	Vermis VI	Left I to IV	T(170) = 6.17	< 0.0001	< 0.0001
	Left Crus I	Left VI	T(170) = 12.83	< 0.0001	< 0.0001
	Right Crus I	Right VI	T(170) = 10.98	< 0.0001	< 0.0001
	Left Crus I	Right VI	T(170) = 9.22	< 0.0001	< 0.0001
	Left Crus I	Vermis VI	T(170) = 8.19	< 0.0001	< 0.0001
	Right Crus I	Vermis VI	T(170) = 7.88	< 0.0001	< 0.0001
	Right Crus I	Left VI	T(170) = 7.47	< 0.0001	< 0.0001
	Left Crus II	Left VI	T(170) = 6.16	< 0.0001	< 0.0001
	Left Crus II	Vermis VI	T(170) = 5.88	< 0.0001	< 0.0001
	Right Crus II	Vermis VI	T(170) = 4.69	< 0.0001	< 0.0001
	Right Crus II	Right VI	T(170) = 4.61	< 0.0001	< 0.0001
	Left Crus II	Right VI	T(170) = 3.22	0.0015	0.0043
	Right Crus II	Left VI	T(170) = 2.06	0.0405	0.0675
	Right putamen	Left pallidum	T(170) = 6.04	< 0.0001	< 0.0001
	Left putamen	Left pallidum	T(170) = 6.10	< 0.0001	< 0.0001
	Right putamen	Right pallidum	T(170) = 5.87	< 0.0001	< 0.0001
	Left putamen	Right pallidum	T(170) = 4.06	< 0.0001	0.0001
	Left caudate	Left pallidum	T(170) = 3.28	0.0006	0.0020
	Left caudate	Right pallidum	T(170) = 2.06	0.0413	0.0688
	Left accumbens	Left pallidum	T(170) = 1.99	0.0483	0.0882
	Vermis Crus II	Vermis VI	T(170) = 6.90	< 0.0001	< 0.0001
	Vermis Crus II	Left VI	T(170) = 6.07	< 0.0001	< 0.0001
	Vermis Crus II	Right VI	T(170) = 4.97	< 0.0001	< 0.0001
	Left pallidum	Left VI	T(170) = 4.15	< 0.0001	< 0.0001
	Right pallidum	Right VI	T(170) = 3.69	0.0001	< 0.0001
	Right pallidum	Left VI	T(170) = 3.59	0.0004	0.0003
	Left pallidum	Right VI	T(170) = 3.13	0.0020	0.0015
	Right pallidum	Vermis VI	T(170) = 2.83	0.0053	0.0176
	Left pallidum	Vermis VI	T(170) = 2.47	0.0143	0.0280
	Left Crus I	Left V	T(170) = 6.57	< 0.0001	< 0.0001
	Left Crus I	Right V	T(170) = 6.27	< 0.0001	< 0.0001
	Right Crus I	Right V	T(170) = 5.75	< 0.0001	< 0.0001
	Right Crus I	Left V	T(170) = 5.66	< 0.0001	< 0.0001
	Right Crus I	Left I to IV	T(170) = 5.17	< 0.0001	< 0.0001
	Right Crus I	Right I to IV	T(170) = 4.96	< 0.0001	< 0.0001
	Left Crus I	Left I to IV	T(170) = 4.86	< 0.0001	< 0.0001
	Left Crus I	Right I to IV	T(170) = 4.57	< 0.0001	< 0.0001
	Left Crus II	Left V	T(170) = 3.48	0.0006	0.0021
	Right Crus II	Right I to IV	T(170) = 3.05	0.0026	0.0068
	Right Crus II	Right V	T(170) = 3.04	0.0027	0.0068
	Left Crus II	Left I to IV	T(170) = 3.00	0.0032	0.0077
	Left Crus II	Right V	T(170) = 2.96	0.0034	0.0077
	Right Crus II	Left I to IV	T(170) = 2.66	0.0085	0.0190
	Left Crus II	Right I to IV	T(170) = 2.50	0.0132	0.0265
	Right Crus II	Left V	T(170) = 2.36	0.0196	0.0356
	Left pallidum	Right pallidum	T(170) = 5.36	< 0.0001	< 0.0001
	Vermis Crus II	Left V	T(170) = 4.74	0.000002	< 0.0001
	Vermis Crus II	Right V	T(170) = 4.32	0.000013	0.0001
	Vermis Crus II	Left I to IV	T(170) = 4.16	0.000025	0.0001
	Vermis Crus II	Right I to IV	T(170) = 3.52	0.0006	0.0014
	Left pallidum	Left I to IV	T(170) = 3.18	0.0017	0.0057
	Left pallidum	Right I to IV	T(170) = 2.98	0.0033	0.0083
	Left pallidum	Right V	T(170) = 2.86	0.0048	0.0107
	Right pallidum	Right I to IV	T(170) = 2.74	0.0068	0.0196
	Left pallidum	Left V	T(170) = 2.45	0.0154	0.0280
	Right pallidum	Right V	T(170) = 2.55	0.0116	0.0290
	Vermis Crus II	Left Crus I	T(170) = 4.23	< 0.0001	0.0001
	Vermis Crus II	Left Crus II	T(170) = 2.19	0.0300	0.0599
	Vermis Crus II	Right Crus I	T(170) = 2.01	0.0457	0.0703
	Right caudate	Right Crus II	T(170) = 3.85	0.0002	0.0006
	Right accumbens	Right Crus II	T(170) = 2.41	0.0170	0.0483
	Left putamen	Left VI	T(170) = 2.03	0.0436	0.0968
**Negative**	Left accumbens	Vermis Crus II	T(170) = −2.40	0.0174	0.0497
	Left caudate	Vermis Crus II	T(170) = −2.10	0.0369	0.0671
	Right caudate	Left pallidum	T(170) = −2.10	0.0373	0.0746
	Right caudate	Vermis Crus II	T(170) = −2.04	0.0433	0.0787
	Right putamen	Left Crus I	T(170) = −2.88	0.0045	0.1115
	Left accumbens	Left Crus I	T(170) = −2.80	0.0057	0.0191
	Left Accumbens	Right Crus I	T(170) = −2.32	0.0215	0.0538
	Right putamen	Right Crus I	T(170) = −2.45	0.0154	0.0343
	Left putamen	Right Crus I	T(170) = −2.60	0.0102	0.0256
	Right putamen	Left Crus II	T(170) = −2.02	0.0452	0.0903
	Right caudate	Left VI	T(170) = −3.58	0.0004	0.0013
	Left caudate	Left VI	T(170) = −3.04	0.0028	0.0079
	Left caudate	Right VI	T(170) = −3.00	0.0031	0.0079
	Left caudate	Vermis VI	T(170) = −2.47	0.0146	0.0291
	Right caudate	Right VI	T(170) = −2.35	0.0199	0.0441
	Left accumbens	Vermis VI	T(170) = −2.06	0.0413	0.0882
	Right accumbens	Left VI	T(170) = −2.57	0.0109	0.0363
	Left caudate	Left V	T(170) = −2.56	0.0114	0.0253
	Right caudate	Left V	T(170) = −2.51	0.0130	0.0326
	Right accumbens	Left V	T(170) = −2.35	0.0200	0.0492
	Right accumbens	Right V	T(170) = −2.22	0.0279	0.0620

Connectivity results were considered significant at a connection-level threshold of p < 0.05 and a cluster-level threshold of pFDR < 0.05 (MVPA omnibus test).

**Table 2.** Lobule V whole brain connectivity in Parkinson’s disease: unadjusted and medication-adjusted models.

Condition	Regions	Cluster (x,y,z)	Size	Peaks	TFCE	*peak p-FDR*
**Without Medication**	Right Crus I	+30 −42 −32	58239	60	903802.83	*< 0.0001*
	Medial/superior frontal gyrus	+06 +40 +42	92635	281	24913.36	*< 0.0001*
	Left precenral gyrus	−26 −22 +72	2316	15	3058.82	*< 0.0001*
	Left inferior temporal gyrus	−50 +00 −48	20	2	826.39	*0.0016*
**Controlling for Medication**	Right VI	+28 −40 −32	33256	63	73179.78	*< 0.0001*
	Medial superior frontal gyrus	−02 +34 +44	28716	164	3365.14	*< 0.0001*
	Left lateral occipital cortex	−38 −76 +38	2591	19	1591.62	*< 0.0001*
	Right lateral occipital cortex	+46 −70 +42	3055	11	1431.61	*0.0001*
	Precuneus/posterior cingulate gyrus	+06 −52 +38	1381	8	1263.80	*0.0004*
	Right anterior temporal pole	+34 +10 −40	3152	29	1259.00	*0.0004*
	Cingulate gyrus, anterior division	+04 +04 +30	38	3	864.54	*0.0024*
	Right postcentral gyrus	+52 −24 +58	47	4	861.70	*0.0024*

Connectivity results were considered significant at a connection-level threshold of p < 0.05 and a cluster-level threshold of *pFDR < 0.05* (MVPA omnibus test).

**Table 3.** Region of Interest (ROI) to ROI association between age and connectivity adjusted for dopaminergic medication.

Region 1	Region 2	Statistic	*p-unc*	*p-FDR*
Left accumbens	Right I to IV	T(169) = −3.70	0.0001	0.0029
Right putamen	Left V	T(169) = −3.42	0.0004	0.0045
Right putamen	Right V	T(169) = −3.38	0.0005	0.0045
Left accumbens	Left I to IV	T(169) = −2.89	0.0022	0.0203
Left caudate	Right V	T(169) = −2.70	0.0038	0.0209
Right accumbens	Right I to IV	T(169) = −2.85	0.0025	0.0249
Left putamen	Right V	T(169) = −2.93	0.0019	0.0329
Left putamen	Left V	T(169) = −2.64	0.0045	0.0329
Right accumbens	Left I to IV	T(169) = −2.58	0.0054	0.0361
Left caudate	Left V	T(169) = −2.14	0.0170	0.0363
Right putamen	Left I to IV	T(169) = −1.98	0.0245	0.0377
Left caudate	Right I to IV	T(169) = −1.98	0.0245	0.0409
Right caudate	Right V	T(169) = −2.21	0.0142	0.0472
Right caudate	Left V	T(169) = −2.11	0.0183	0.0506
Right accumbens	Left V	T(169) = −2.26	0.0126	0.0570
Right accumbens	Right V	T(169) = −1.94	0.0268	0.0767
Left accumbens	Right V	T(169) = −1.84	0.0337	0.0857

Connectivity results were considered significant at a connection-level threshold of p < 0.05 and a cluster-level threshold of pFDR < 0.05 (MVPA omnibus test).

**Table 4.** Region of Interest (ROI) to ROI effects of disease duration on connectivity adjusted for dopaminergic medication, with separate sections demonstrating for whole participants, cognitively normal PD, and PD with cognitive dysfunction.

Group	Region 1	Region 2	Statistic	p-unc	p-FDR
**All PD**	Right I to IV	Left I to IV	T(169) = 6.92	< 0.0001	< 0.0001
	Right V	Left V	T(169) = 5.52	< 0.0001	< 0.0001
	Right V	Left I to IV	T(169) = 5.04	< 0.0001	< 0.0001
	Right V	Right I to IV	T(169) = 4.96	< 0.0001	< 0.0001
	Left V	Left I to IV	T(169) = 4.49	< 0.0001	0.0001
	Left V	Right I to IV	T(169) = 4.44	< 0.0001	0.0001
	Left pallidum	Right pallidum	T(169) = 5.94	< 0.0001	< 0.0001
	Right pallidum	Vermis Curs II	T(169) = 2.72	0.0073	0.0488
	Right putamen	Left putamen	T(169) = 4.50	< 0.0001	0.0003
	Right caudate	Left caudate	T(169) = 3.28	0.0013	0.0250
	Right accumbens	Left accumbens	T(169) = 2.34	0.0202	0.1458
	Right caudate	Right accumbens	T(169) = −2.47	0.0146	0.1460
	Left Crus II	Right Crus II	T(169) = 4.38	< 0.0001	0.0004
	Right Crus I	Left Crus I	T(169) = 2.04	0.0433	0.2167
	Left Crus II	Right Crus I	T(169) = 2.04	0.0426	0.2840
	Right VI	Left V	T(169) = 3.47	0.0006	0.0065
	Right VI	Right I to IV	T(169) = 2.78	0.0060	0.0401
	Right VI	Left I to IV	T(169) = 2.68	0.0081	0.0404
	Right VI	Right V	T(169) = 2.17	0.0312	0.1041
	Left VI	Left V	T(169) = 2.25	0.0260	0.1555
	Left VI	Right I to IV	T(169) = 2.17	0.0313	0.1555
	Vermis Crus II	Vermis VI	T(169) = 2.69	0.0079	0.0393
	Right pallidum	Vermis VI	T(169) = 2.76	0.0064	0.0488
	Right pallidum	Right VI	T(169) = 2.46	0.0151	0.0754
	Right VI	Left VI	T(169) = 3.93	0.0001	0.0025
	Right putamen	Left pallidum	T(169) = 3.75	0.0002	0.0025
	Right accumbens	Right pallidum	T(169) = 2.29	0.0231	0.1458
	Right accumbens	Left pallidum	T(169) = 2.20	0.0291	0.1458
	Left accumbens	Left pallidum	T(169) = 2.68	0.0081	0.1615
	Left accumbens	Right pallidum	T(169) = 2.21	0.0283	0.1886
	Left putamen	Right pallidum	T(169) = 2.12	0.0357	0.2377
**PD: cognitively normal**	Right I to IV	Left I to IV	T(102) = 6.19	< 0.0001	< 0.0001
	Right V	Left V	T(102) = 5.55	< 0.0001	< 0.0001
	Right V	Right I to IV	T(102) = 5.46	< 0.0001	< 0.0001
	Left V	Right I to IV	T(102) = 5.14	< 0.0001	< 0.0001
	Right V	Left I to IV	T(102) = 4.49	< 0.0001	0.0001
	Left V	Left I to IV	T(102) = 4.45	< 0.0001	0.0001
	Left pallidum	Right pallidum	T(102) = 4.64	< 0.0001	0.0002
	Right pallidum	Vermis Crus II	T(102) = 2.22	0.0290	0.2897
	Right putamen	Left putamen	T(102) = 3.15	0.0022	0.0296
	Right caudate	Right accumbens	T(102) = −2.61	0.0104	0.1740
	Right caudate	Left caudate	T(102) = 2.42	0.0174	0.1740
	Left Crus II	Right Crus II	T(102) = 4.34	< 0.0001	0.0007
	Right Crus I	Left Crus I	T(102) = 2.14	0.0350	0.2334
**PD: cognitive decline**	Left VI	Left V	T(64) = 2.39	0.0200	0.1331
	Right VI	Right V	T(64) = 2.22	0.0299	0.1990
	Left Pallidum	Right pallidum	T(64) = 3.78	0.0004	0.0070

Connectivity results were considered significant at a connection-level threshold of p < 0.05 and a cluster-level threshold of pFDR < 0.05 (MVPA omnibus test).

**Table 5.** Lobule V whole brain connectivity associated with disease duration in Parkinson’s disease adjusted for dopaminergic medication

Region	Cluster (x,y,z)	Size	Peaks	TFCE	peak p-FDR
Vermis IV to VI	−06 −36 −24	7880	60	3120.01	< 0.0001
Right precentral gyrus	+44 +04 +42	702	10	1021.96	0.0112
Left middle frontal gyrus	−28 +08 +34	437	9	995.66	0.0121
Left superior frontal gyrus	−16 +04 +58	132	3	922.70	0.0162
Medial anterior cingulate gyrus	+06 +14 +36	100	1	866.40	0.0179
Left frontal pole	−28 +40 +28	45	1	864.89	0.0179
Right middle frontal gyrus	+30 +16 +24	1	1	844.00	0.0189
Right inferior frontal gyrus/frontal operculum	+32 +16 +20	2	1	841.79	0.0189

Connectivity results were considered significant at a connection-level threshold of p < 0.05 and a cluster-level threshold of pFDR < 0.05 (MVPA omnibus test).

## References

[1] Tanner CM, Ostrem JL. Parkinson's Disease. N Engl J Med. Aug 1 2024;391(5):442–452. doi:10.1056/NEJMra2401857 39083773

[2] Aarsland D, Batzu L, Halliday GM, Parkinson disease-associated cognitive impairment. Nat Rev Dis Primers. Jul 1 2021;7(1):47. doi:10.1038/s41572-021-00280-3 34210995

[3] Saredakis D, Collins-Praino LE, Gutteridge DS, Stephan BCM, Keage HAD. Conversion to MCI and dementia in Parkinson's disease: a systematic review and meta-analysis. Parkinsonism Relat Disord. Aug 2019;65:20–31. doi:10.1016/j.parkreldis.2019.04.020 31109727

[4] Buckner RL. The cerebellum and cognitive function: 25 years of insight from anatomy and neuroimaging. Neuron. Oct 30 2013;80(3):807–15. doi:10.1016/j.neuron.2013.10.044 24183029

[5] Herculano-Houzel S, Lent R. Isotropic fractionator: a simple, rapid method for the quantification of total cell and neuron numbers in the brain. J Neurosci. Mar 9 2005;25(10):2518–21. doi:10.1523/jneurosci.4526-04.2005 15758160 PMC6725175

[6] Magielse N, Heuer K, Toro R, Schutter D, Valk SL. A Comparative Perspective on the Cerebello-Cerebral System and Its Link to Cognition. Cerebellum. Dec 2023;22(6):1293–1307. doi:10.1007/s12311-022-01495-0 36417091 PMC10657313

[7] Arleo A, Bareš M, Bernard JA, Consensus Paper: Cerebellum and Ageing. Cerebellum. Apr 2024;23(2):802–832. doi:10.1007/s12311-023-01577-7 37428408 PMC10776824

[8] Rudolph S, Badura A, Lutzu S, Cognitive-Affective Functions of the Cerebellum. J Neurosci. Nov 8 2023;43(45):7554–7564. doi:10.1523/jneurosci.1451-23.2023 37940582 PMC10634583

[9] Li T, Le W, Jankovic J. Linking the cerebellum to Parkinson disease: an update. Nat Rev Neurol. Nov 2023;19(11):645–654. doi:10.1038/s41582-023-00874-3 37752351

[10] Festini SB, Bernard JA, Kwak Y, Altered cerebellar connectivity in Parkinson's patients ON and OFF L-DOPA medication. Front Hum Neurosci. 2015;9:214. doi:10.3389/fnhum.2015.00214 25954184 PMC4405615

[11] Jiang L, Zhuo J, Furman A, Fishman PS, Gullapalli R. Cerebellar functional connectivity change is associated with motor and neuropsychological function in early stage drug-naïve patients with Parkinson's disease. Front Neurosci. 2023;17:1113889. doi:10.3389/fnins.2023.1113889 37425003 PMC10324581

[12] Kerestes R, Laansma MA, Owens-Walton C, Cerebellar Volume and Disease Staging in Parkinson's Disease: An ENIGMA-PD Study. Mov Disord. Dec 2023;38(12):2269–2281. doi:10.1002/mds.29611 37964373 PMC10754393

[13] O'Callaghan C, Hornberger M, Balsters JH, Halliday GM, Lewis SJ, Shine JM. Cerebellar atrophy in Parkinson's disease and its implication for network connectivity. Brain. Mar 2016;139(Pt 3):845–55. doi:10.1093/brain/awv399 26794597

[14] He C, Yang R, Rong S, Temporal evolution of microstructural integrity in cerebellar peduncles in Parkinson's disease: Stage-specific patterns and dopaminergic correlates. Neuroimage Clin. 2024;44:103679. doi:10.1016/j.nicl.2024.103679 39366283 PMC11489329

[15] Palmer WC, Cholerton BA, Zabetian CP, Montine TJ, Grabowski TJ, Rane S. Resting-State Cerebello-Cortical Dysfunction in Parkinson's Disease. Front Neurol. 2020;11:594213. doi:10.3389/fneur.2020.594213 33584497 PMC7876057

[16] Seidel K, Bouzrou M, Heidemann N, Involvement of the cerebellum in Parkinson disease and dementia with Lewy bodies. Ann Neurol. Jun 2017;81(6):898–903. doi:10.1002/ana.24937 28439961

[17] Qiu T, Liu M, Qiu X, Li T, Le W. Cerebellar involvement in Parkinson's disease: Pathophysiology and neuroimaging. Chin Med J (Engl). Oct 20 2024;137(20):2395–2403. doi:10.1097/cm9.0000000000003248 39227357 PMC11479504

[18] Wu T, Hallett M. The cerebellum in Parkinson's disease. Brain. Mar 2013;136(Pt 3):696–709. doi:10.1093/brain/aws360 23404337 PMC7273201

[19] Xu S, He XW, Zhao R, Cerebellar functional abnormalities in early stage drug-naïve and medicated Parkinson's disease. J Neurol. Jul 2019;266(7):1578–1587. doi:10.1007/s00415-019-09294-0 30923933

[20] Bernard JA. Don't forget the little brain: A framework for incorporating the cerebellum into the understanding of cognitive aging. Neurosci Biobehav Rev. Jun 2022;137:104639. doi:10.1016/j.neubiorev.2022.104639 35346747 PMC9119942

[21] Bernard JA, McOwen KM, Huynh AT. New Frontiers for the Understanding of Aging: The Power and Possibilities of Studying the Cerebellum. Curr Opin Behav Sci. Dec 2023;54 doi:10.1016/j.cobeha.2023.101311 PMC10939048 38496767

[22] Iskusnykh IY, Zakharova AA, Kryl'skii ED, Popova TN. Aging, Neurodegenerative Disorders, and Cerebellum. Int J Mol Sci. Jan 13 2024;25(2)doi:10.3390/ijms25021018 PMC10815822 38256091

[23] Liu G, Yang C, Wang X, Chen X, Cai H, Le W. Cerebellum in neurodegenerative diseases: Advances, challenges, and prospects. iScience. Nov 15 2024;27(11):111194. doi:10.1016/j.isci.2024.111194 39555407 PMC11567929

[24] Mitoma H, Buffo A, Gelfo F, Consensus Paper. Cerebellar Reserve: From Cerebellar Physiology to Cerebellar Disorders. Cerebellum. Feb 2020;19(1):131–153. doi:10.1007/s12311-019-01091-9 31879843 PMC6978437

[25] Hausman HK, Jackson TB, Goen JRM, Bernard JA. From Synchrony to Asynchrony: Cerebellar-Basal Ganglia Functional Circuits in Young and Older Adults. Cereb Cortex. Mar 21 2020;30(2):718–729. doi:10.1093/cercor/bhz121 31219563

[26] Kelly C, de Zubicaray G, Di Martino A, L-dopa modulates functional connectivity in striatal cognitive and motor networks: a double-blind placebo-controlled study. J Neurosci. Jun 3 2009;29(22):7364–78. doi:10.1523/jneurosci.0810-09.2009 19494158 PMC2928147

[27] Marek K. The Parkinson's Progression Markers Inititiative (PPMI) Clinical-Establishing a Deeply Phenotyped PD Cohort AM 4 V.3. protocols.io 2024. 10.17504/protocols.io.n92ldmw6ol5b/v2

[28] Jones JD, Kuhn TP, Szymkowicz SM. Reverters from PD-MCI to cognitively intact are at risk for future cognitive impairment: Analysis of the PPMI cohort. Parkinsonism Relat Disord. Feb 2018;47:3–7. doi:10.1016/j.parkreldis.2017.12.006 29233608 PMC5803409

[29] MaldonadoT, JacksonTB, RezaeeZ, BernardJA. Time Dependent Effects of Cerebellar tDCS on Cerebello-cortical Connectivity Networks in Young Adults. Cerebellum. Jan 10 2025;24(1):29. doi:10.1007/s12311-024-01781-z39794631

[30] Whitfield-Gabrieli S, Nieto-Castanon A. Conn: a functional connectivity toolbox for correlated and anticorrelated brain networks. Brain Connect. 2012;2(3):125–41. doi:10.1089/brain.2012.0073 22642651

[31] Nieto-Castanon A. Handbook of functional connectivity Magnetic Resonance Imaging methods in CONN. Hilbert Press; 2020.

[32] Andersson JL, Hutton C, Ashburner J, Turner R, Friston K. Modeling geometric deformations in EPI time series. Neuroimage. May 2001;13(5):903–19. doi:10.1006/nimg.2001.0746 11304086

[33] Friston KJ, Ashburner J, Frith CD, Poline J-B, Heather JD, Frackowiak RSJ. Spatial registration and normalization of images. Human Brain Mapping. 1995;3(3):165–189. doi:10.1002/hbm.460030303

[34] Power JD, Mitra A, Laumann TO, Snyder AZ, Schlaggar BL, Petersen SE. Methods to detect, characterize, and remove motion artifact in resting state fMRI. Neuroimage. Jan 1 2014;84:320–41. doi:10.1016/j.neuroimage.2013.08.048 23994314 PMC3849338

[35] Ashburner J. A fast diffeomorphic image registration algorithm. Neuroimage. Oct 15 2007;38(1):95–113. doi:10.1016/j.neuroimage.2007.07.007 17761438

[36] Ashburner J, Friston KJ. Unified segmentation. Neuroimage. Jul 1 2005;26(3):839–51. doi:10.1016/j.neuroimage.2005.02.018 15955494

[37] Calhoun VD, Wager TD, Krishnan A, The impact of T1 versus EPI spatial normalization templates for fMRI data analyses. Hum Brain Mapp. Nov 2017;38(11):5331–5342. doi:10.1002/hbm.23737 28745021 PMC5565844

[38] Friston KJ, Williams S, Howard R, Frackowiak RS, Turner R. Movement-related effects in fMRI time-series. Magn Reson Med. Mar 1996;35(3):346–55. doi:10.1002/mrm.1910350312 8699946

[39] Hallquist MN, Hwang K, Luna B. The nuisance of nuisance regression: spectral misspecification in a common approach to resting-state fMRI preprocessing reintroduces noise and obscures functional connectivity. Neuroimage. Nov 15 2013;82:208–25. doi:10.1016/j.neuroimage.2013.05.116 23747457 PMC3759585

[40] Behzadi Y, Restom K, Liau J, Liu TT. A component based noise correction method (CompCor) for BOLD and perfusion based fMRI. Neuroimage. Aug 1 2007;37(1):90–101. doi:10.1016/j.neuroimage.2007.04.042 17560126 PMC2214855

[41] Diedrichsen J. A spatially unbiased atlas template of the human cerebellum. Neuroimage. Oct 15 2006;33(1):127–38. doi:10.1016/j.neuroimage.2006.05.056 16904911

[42] Diedrichsen J, Balsters JH, Flavell J, Cussans E, Ramnani N. A probabilistic MR atlas of the human cerebellum. Neuroimage. May 15 2009;46(1):39–46. doi:10.1016/j.neuroimage.2009.01.045 19457380

[43] Buckner RL, Krienen FM, Castellanos A, Diaz JC, Yeo BT. The organization of the human cerebellum estimated by intrinsic functional connectivity. J Neurophysiol. Nov 2011;106(5):2322–45. doi:10.1152/jn.00339.2011 21795627 PMC3214121

[44] Krienen FM, Buckner RL. Segregated fronto-cerebellar circuits revealed by intrinsic functional connectivity. Cereb Cortex. Oct 2009;19(10):2485–97. doi:10.1093/cercor/bhp135 19592571 PMC2742600

[45] O'Reilly JX, Beckmann CF, Tomassini V, Ramnani N, Johansen-Berg H. Distinct and overlapping functional zones in the cerebellum defined by resting state functional connectivity. Cereb Cortex. Apr 2010;20(4):953–65. doi:10.1093/cercor/bhp157 19684249 PMC2837094

[46] Smith SM, Nichols TE. Threshold-free cluster enhancement: addressing problems of smoothing, threshold dependence and localisation in cluster inference. Neuroimage. Jan 1 2009;44(1):83–98. doi:10.1016/j.neuroimage.2008.03.061 18501637

[47] Ballard HK, Jackson TB, Hicks TH, Bernard JA. The association of reproductive stage with lobular cerebellar network connectivity across female adulthood. Neurobiol Aging. Sep 2022;117:139–150. doi:10.1016/j.neurobiolaging.2022.05.014 35738086 PMC10149146

[48] Bernard JA, Peltier SJ, Wiggins JL, Disrupted cortico-cerebellar connectivity in older adults. Neuroimage. Dec 2013;83:103–19. doi:10.1016/j.neuroimage.2013.06.042 23792980 PMC3815977

[49] Bernard JA, Seidler RD, Hassevoort KM, Resting state cortico-cerebellar functional connectivity networks: a comparison of anatomical and self-organizing map approaches. Front Neuroanat. 2012;6:31. doi:10.3389/fnana.2012.00031 22907994 PMC3415673

[50] Bernard JA, Nguyen AD, Hausman HK, Shaky scaffolding: Age differences in cerebellar activation revealed through activation likelihood estimation meta-analysis. Hum Brain Mapp. Dec 15 2020;41(18):5255–5281. doi:10.1002/hbm.25191 32936989 PMC7670650

[51] Bernard JA, Seidler RD. Moving forward: age effects on the cerebellum underlie cognitive and motor declines. Neurosci Biobehav Rev. May 2014;42:193–207. doi:10.1016/j.neubiorev.2014.02.011 24594194 PMC4024443

[52] Mitoma H, Kakei S, Yamaguchi K, Manto M. Physiology of Cerebellar Reserve: Redundancy and Plasticity of a Modular Machine. Int J Mol Sci. Apr 30 2021;22(9)doi:10.3390/ijms22094777 PMC8124536 33946358

[53] Cabeza R, Albert M, Belleville S, Maintenance, reserve and compensation: the cognitive neuroscience of healthy ageing. Nat Rev Neurosci. Nov 2018;19(11):701–710. doi:10.1038/s41583-018-0068-2 30305711 PMC6472256

[54] Liang KJ, Carlson ES. Resistance, vulnerability and resilience: A review of the cognitive cerebellum in aging and neurodegenerative diseases. Neurobiol Learn Mem. Apr 2020;170:106981. doi:10.1016/j.nlm.2019.01.004 30630042 PMC6612482

